# Delayed Migration of a WallFlex Enteral Stent Resulting in Jejunal Perforation

**DOI:** 10.1155/2013/652597

**Published:** 2013-04-22

**Authors:** Phillip S. Ge, Rabindra R. Watson, David C. Chen, V. Raman Muthusamy

**Affiliations:** ^1^Department of Medicine, UCLA Medical Center, Los Angeles, CA 90095, USA; ^2^Division of Digestive Diseases, UCLA Medical Center, Los Angeles, CA 90095, USA; ^3^Department of Surgery, UCLA Medical Center, Los Angeles, CA 90095, USA

## Abstract

Enteral stents are increasingly utilized to palliate malignant gastrointestinal obstruction; however, they can be associated with significant complications. We describe an unusual case of a 67-year-old male with gastric adenocarcinoma who underwent placement of a WallFlex metallic enteral stent to relieve a malignant gastric outlet obstruction. Four months later, while actively undergoing chemotherapy, he developed acute abdominal pain and was found to have delayed stent migration and jejunal perforation. He required emergent surgical resection of the perforated segment of jejunum. Delayed migration of the WallFlex enteral stent with subsequent visceral perforation has yet to be reported in the literature. Chemotherapy after stent placement has been associated with an increase in maintenance of stent patency; however, shrinkage of the local tumor by chemoradiation may increase the risk of stent migration. Care should be taken in placing enteral stents in patients undergoing continued treatment of their malignancy, as delayed migration of even uncovered stents may occur.

## 1. Introduction

Endoscopic enteral stent placement is commonly performed for palliative treatment of malignant gastric outlet obstruction [1]. This minimally invasive procedure, first described in 1992, involves the trans-endoscopic placement of a self-expandable uncovered metallic stent across a malignant obstruction [2, 3]. A number of studies have since demonstrated the safety and feasibility of this technique [4–9]. However, enteral stents can occasionally be associated with significant complications including perforation, bleeding, stent migration (immediate or delayed), stent malposition, and stent occlusion from tumor growth or food impaction [1]. The combination of delayed stent migration and perforation is extremely rare [9, 10]. Here we describe a case of delayed stent migration and jejunal perforation occurring four months after the insertion of a WallFlex metallic enteral stent to relieve malignant gastric outlet obstruction.

## 2. Case Report

**Figure 1:**
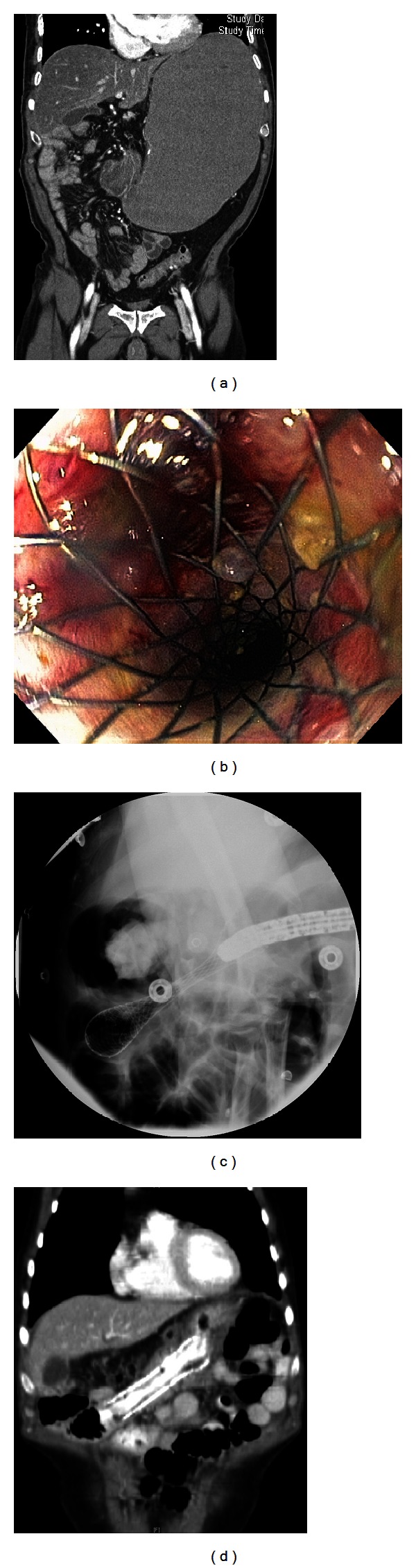
(a) Coronal CT image reveals a malignant gastric outlet obstruction at the gastrojejunal anastomosis. (b) The patient underwent endoscopic placement of a 90 mm × 22 mm WallFlex Enteral Stent across the malignant stricture. (c) Fluoroscopy immediately following stent placement confirms the enteral stent in place. (d) Coronary CT image obtained two months following stent placement confirms the enteral stent in place.

A 67-year-old male with stage IIIB gastric adenocarcinoma treated with Billroth-II gastrojejunostomy, adjuvant chemotherapy with oxaliplatin and 5-fluorouracil, and external beam radiation therapy presented one year following completion of adjuvant therapy with abdominal pain and inability to tolerate oral intake. Imaging studies including computed tomography (CT) of the abdomen and pelvis revealed a malignant gastric outlet obstruction at the gastrojejunal anastomosis (Figure 1(a)). Following nasogastric decompression, he underwent endoscopic placement of a 90 mm × 22 mm WallFlex Enteral Stent (Boston Scientific, Natick, MA) across the stricture (Figures 1(b)–1(d)). There were no immediate complications, his abdominal pain resolved, and he resumed oral intake. He was initiated on docetaxel therapy and discharged home.

**Figure 2:**
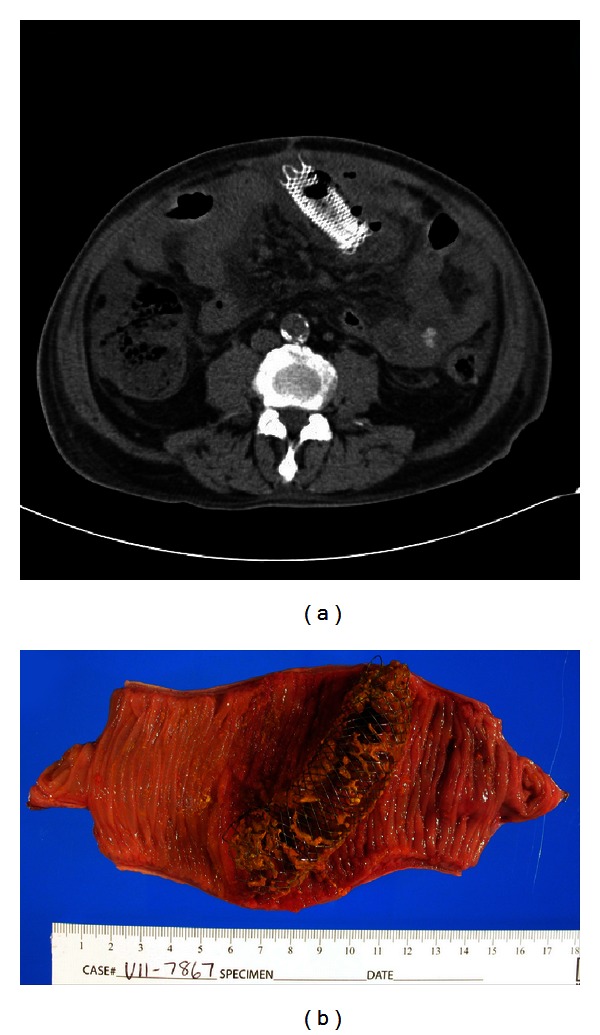
(a) Axial CT image showing migration of the stent distally into the mid-jejunum, with resultant visceral perforation. (b) Surgical specimen showing dislodged enteral stent with perforation of the mid-jejunum.

Four months later, after four cycles of docetaxel, he presented with severe acute abdominal pain with peritoneal signs. Laboratories revealed white blood cell count of 17,720/*μ*L (reference: 3.28–9.29 × 10^3^/*μ*L) with 93.0% neutrophils. CT imaging (Figure 2(a)) showed migration of the stent 20 cm distally into the mid-jejunum, resulting in visceral perforation, stent erosion into the anterior abdominal wall, leakage of enteric contents, and peritonitis. He emergently underwent operative resection of the perforated segment of jejunum (Figure 2(b)) with primary re-anastomosis. His postoperative course was unremarkable and he was discharged home tolerating a regular diet. He has subsequently done well on continued chemotherapy.

## 3. Discussion

Gastric outlet obstruction is a feared late complication of unresectable periampullary, distal gastric, pancreatic, and duodenal malignancy. Symptoms of gastric outlet obstruction include intractable nausea and vomiting, cachexia, and a progressive decline in clinical status and in quality of life [9]. Endoscopic enteral stent placement is becoming increasingly utilized for the minimally invasive palliative treatment of malignant gastrointestinal obstructions, with success rates favorable to surgical bypass [11]. A prospective randomized trial comparing laparoscopic gastrojejunostomy versus duodenal stenting for malignant gastric outlet obstruction showed that duodenal stenting provided a superior means of palliation compared to laparoscopic gastrojejunostomy, with decreased in-hospital complications, decreased pain, and increased quality of life, without significant difference in cumulative survival [12]. The reported technical success rate of endoscopic stenting is approximately 94–97%, with clinical success rates of 87–94%; patients are typically able to resume oral intake within 4 days [8, 13, 14].

Despite widespread use, severe complications of perforation and bleeding have only been reported in 0.7% and 0.5% of patients, respectively [8]. Nonsevere complications (stent obstruction, stent migration, pain, etc.) have been reported in 26.7% of patients, including stent migration in 5.1% [8]. Most of the existing published data reflect experience with the enteral Wallstent (Boston Scientific, Natick, MA) [7–9, 14, 15]. The limited flexibility of the Wallstent's metal wire mesh can contribute to stent migration, and the sharp ends can cause mucosal ulceration with subsequent risk of bleeding and perforation [10]. The newer WallFlex stent has a nickel titanium alloy mesh to improve flexibility while maintaining lumen integrity, looped ends to reduce the risk of mucosal injury, and a proximal flared end to minimize the risk of stent migration [13].

Prospective studies involving the WallFlex stent have only reported rare incidences of stent migration or perforation within the first month following placement [13, 16]. Delayed migration of the WallFlex stent with visceral perforation has yet to be reported in the literature. Chemotherapy following stent placement has been associated with an increase in maintenance of stent patency [7, 17]; however, shrinkage of the local tumor by chemoradiation may increase the risk of stent migration [18]. This may be due to a reduction in luminal compression resulting in decreased contact of the intestinal wall with the stent, or to regression of tissue overgrowth of the mesh of the stent, leading to dislodgment of the device. Subsequent mechanical shear of the stent against the intestinal mucosa may cause ulceration and perforation.

As this case illustrates, care should be taken in placing enteral stents in patients undergoing continued treatment of their malignancy, as delayed migration of even uncovered stents may occur. When evaluating a patient with a history of an enteral stent presenting with acute abdominal pain, it is important to maintain a high degree of suspicion for stent migration resulting in either visceral perforation and/or intestinal obstruction. Immediate imaging should be undertaken to rule out stent-related complications, even if several months have elapsed since stent placement.
